# Letter from the new Editor-in-Chief for Insights into Imaging

**DOI:** 10.1007/s13244-018-0617-2

**Published:** 2018-03-28

**Authors:** Luis Martí-Bonmatí

**Affiliations:** Medical Imaging Department and Biomedical Imaging Research Group (GIBI230), La Fe Polytechnics and University Hospital and Health Research Institute, Valencia, Spain

## Critical thinking from education to innovation in medical imaging

When the European Society of Radiology (ESR) announced my appointment as the new Editor-in-Chief of *Insights into Imaging* starting from January 2018, Prof. Robert Hermans, who has served as the Editor-in-Chief since 2010, continued to provide support throughout the transition period. During his editorship, the journal has developed into a very successful international open-access peer-reviewed journal. The educational journal is appreciated all over the world as a high-quality and up-to-date source of information in the field of radiology.

## The journal’s content

At *Insights into Imaging*, we want to provide relevant professional knowledge as an addition to the clinical and research-focused content covered by the other members of the ESR journal family, *European Radiology* and *European Radiology Experimental*.

*Insights into Imaging* will largely focus on publishing in-depth critical reviews and illustrated pictorial articles, helping radiologists to envision the best way to meet patients’ needs while improving healthcare performance. Furthermore, the journal is also looking for submissions from leading European radiological societies and groups willing to improve the organisation of radiological departments and to strengthen relationships with other hospital departments. In addition, we very much welcome relevant papers dealing with standardisation proposals, state-of-the-art topics, research critiques and opportunities and the future of medical imaging. *Insights into Imaging* is meant to inspire healthcare professionals to develop expert opinions and take on new challenges.

## The readers

We recognise that the greatest challenge for our journal is to strengthen the connection with the readership. As a high-quality platform for the dissemination of critical and useful knowledge, the journal will aid in the transformation of radiological practice towards an evidence-based, technology-based and patient-driven focus. To succeed as a journal, the topics covered have to be constantly exciting and relevant. The purpose of a scientific paper is to analyse and dissect a whole into parts to discover its nature, function and context, and transform it into knowledge through logical reasoning. *Insights into Imaging* supports radiologists by discussing topics such as how to properly perform exams, how to report and how to measure and quantify imaging data and tissue properties. The journal provides input on what we know and explores what we should know to improve the healthcare cycle through the use of medical imaging and related computer science.

## The authors

Having been involved in academia and research, and with previous editorial experience on different Editorial Boards of major radiological journals, I fully recognise that the authors are the most important stakeholders in quality publishing. Authors should be considered as the main clients of our journal.

The journal focuses and strengthens the role of radiologists in healthcare. By publishing critical reviews, up-to-date evaluations, guidelines, topics with the highest level of evidence, professional and management issues, new developments and meta-analyses, *Insights into Imaging* will help readers refine their critical thinking skills and encourage them to develop stronger connections with their colleagues and other healthcare professionals. The most important articles will focus on patient-centred imaging and quality processes.

I would advise authors to focus on reviews and state-of-the-art papers in their field of expertise. Review papers should cover educational aspects of imaging with a pearls-and-pitfalls approach for pictorial reviews or give an overview of emerging techniques and cutting-edge concepts with an up-to-date and innovative approach for critical reviews. Both pictorial and critical reviews have significant potential to serve the radiological community, providing access to the best evidence-based research and displaying the impact of new imaging approaches.

I particularly want to encourage subspecialty societies and networking groups to submit position papers, including imaging guidelines on patient management aspects, as well as consensus statements on imaging-related topics. Furthermore, we will introduce in-depth commentaries of articles recently published in various radiological journals, not only in *Insights into Imaging*.

As a general rule, I advise authors to be original and to develop the critical thinking skills necessary to write any important paper; to choose a well-defined interesting topic on an important aspect related to imaging and to stay focused on it; to search the literature for relevant papers and related reviews; to be up to date but not forget older studies; to take notes while reading; to rewrite, restructure and rethink in order to obtain a text with a coherent argument; to avoid any possible plagiarism; and, finally, to be critical and consistent, identifying methodological problems while pointing out knowledge and research gaps.

As papers are fully available online worldwide, their impact in the community is large and they are easily citable. Outstanding and steadily increasing download numbers prove that publishing in *Insights into Imaging* will ensure excellent visibility of your work and your team. Open-access publishing is not without cost, and I am happy to say that the ESR covers the article processing charges for all corresponding authors who are active society members and have no other funding options.

## The reviewers

In order to attract the best manuscripts and to provide good feedback to authors, high-quality reviews are essential. Reviewers spend much time and effort helping authors to improve the quality of their submitted manuscripts and assisting the Editor-in-Chief in selecting the best papers to be published. Their work is immensely valuable. Therefore, it is important to constantly improve the review process and increase the number of reviewers within the defined areas of interest and expertise. I do invite all of you interested in reviewing to send us an e-mail (office@i3-journal.org) stating your fields of interest and expertise.

## The conclusions

In summary, *Insights into Imaging* is an international, high-quality, peer-reviewed, open-access journal that makes your work immediately and permanently available online, for everyone, worldwide. I hope that we will all further enhance the journal’s quality and standing within the radiological and medical imaging community.

